# In Situ Synthesis of 3D BiOCl–Graphene Aerogel and Synergistic Effect by Photo-Assisted Activation of Persulfate for Methyl Orange Degradation

**DOI:** 10.3390/molecules28134964

**Published:** 2023-06-24

**Authors:** Yukun Li, Dan Zhang, Yongshu Zhang, Cong Chao, Qishi Chen, Sen Yao, Cuixia Liu

**Affiliations:** 1 School of Energy and Environmental Engineering, Zhongyuan University of Technology, Zhengzhou 450007, China; 2 Science and Technology Innovation Coordination Service Center of Laiwu District, Jinan 271100, China

**Keywords:** persulfate, graphene aerogel, photocatalytic activation, BiOCl, simulated sunlight

## Abstract

BiOCl/graphene aerogel graphene (BGA) was successfully obtained by in situ hydrothermal synthesis, and the chemical, structural, morphological, and photocatalytic properties were systematically characterized. BGA with the doping amount of BiOCl at 20% (BGA-4) exhibited the optimal activation efficiency for persulfate (PDS) on the degradation of methyl orange (MO) under simulated sunlight (SSL) illumination as compared to the pure graphene (GA) and aerogel composites with different BiOCl content. The influence of various reaction parameters on the MO removal efficiency, such as the reaction system, catalyst activator dose, PDS concentration, BiOCl doping amount, and the initial pH of the solution, was investigated. Under optimum conditions, the catalytic efficiency of BiOCl-doped GA with the mass ratio of 20% (BGA-4) was 5.61 times that of GA. The strengthening effect of BGA-4 benefited from the synergistic effect of ^1^O_2_, O_2_·^−^ and the generation and rapid electron transfer of photo-induced electron (e^−^) in the BGA-4/SSL/PDS system. Considering the superior stability and recyclability of BGA-4, the BGA-4/SSL/PDS system exhibits great potential in actual wastewater treatment.

## 1. Introduction

Water is the indispensably precious resource that mankind depends on for existence and development [1]. By the year 2025, the number of people who lack access to safe drinking water is estimated to be more than 2 billion; nearly 20% of the global population will be living in countries or regions with absolute water scarcity [2,3]. Unfortunately, rapid economic development, industrialization, and urbanization have exacerbated water shortages and water quality degradation. Particularly, the enrichment of textile printing and dyeing wastewater possesses a remarkable effect on the water ecosystem and food chain. More seriously, azo dye wastewater not only affects the germination and growth of plant seeds but also has carcinogenic, teratogenic, and mutagenic effects on humans and animals health [4]. Thus, increasing studies have placed emphasis on the decomposition of azo dye wastewater through an efficient method in recent years. The traditional treatment methods of azo dye wastewater, such as biodegradation [5], physical adsorption [6], and ozonation [7], have been widely employed in research and development. Recently, many researchers have paid more attention to advanced oxidation processes (AOPs) which degrade azo dye into harmless inorganic matters via redox reaction. Among the reported available AOPs, Fenton or Fenton-like technology is an effective method for degrading refractory organic pollutants. However, the narrow acidic operation pH, unrecyclable catalysts, and the production of iron sludge are the limiting factors for scale application of Fenton or Fenton-like technology [8]. Recently, sulfate radicals (SO_4_**^.^**^−^) activated by persulfate (PS) have attracted extensive attention due to their high redox potential of 2.5–3.1 V, which is comparable to hydroxyl radicals (·OH, 2.8 V) [9]. The oxidation-reduction potential, environmental friendliness, and selectivity for removing refractory organic pollutants attributed to the redox properties of PS make it possible for a viable alternative to replace H_2_O_2_ [10,11]. However, the oxidation property of PS itself is not prominent, and the performance could be strengthened by means of activation [12].

SO_4_·^−^ could be generated efficiently by activating peroxide bonds in PS using external energy (heat, ultrasound, UV irradiation, or microwave), metallic materials (mostly transition metals), and non-metallic materials (carbon-based material materials, quinones, or strong alkaline) [13]. Among these methods, physical activation is energy-intensive, and metal-based catalysts may cause the release of metal ions [14]. Recently, the carbon-based materials have made significant progress in the activation of PS in terms of its low cost and electrical conductivity [15]. Graphene is a honeycomb-like sheet of carbon, just one atom thick, with the excellent features of large specific surface area, high electrical and thermal conductivity, and mechanical properties [16]. It was first reported that reduced graphene oxide (rGO) could activate PMS to generate SO_4_**·**^−^, and the catalytic performances of rGO could be enhanced with the increase in oxygen containing groups [17]. However, the separation of the powdered rGO is time-consuming and costly. The graphene aerogel with three-dimensional (3D) structure prepared from graphene nanosheets by certain method can resolve the catalyst recovery and secondary pollution problems. Previous study by Karbasi et al. [18] showed that macroscopic rGO aerogels played an important role in inducing highly efficient radicals through PMS activation. It is a good choice to construct semiconductor composites based on aerogel. Ma et al. reported that carbon aerogel/TiO_2_ composite material is a promising photocatalysts for environmental pollutants degradation owing to the decreased recombination probability of photo-induced carriers [19]. The abundant pore structure and large surface area of 3D graphene aerogel make semiconductor materials uniformly disperse on the surface of graphene. The black body and surface sensitization would be conducive to promote the visible optical absorption and the charge transportation and separation. Cu_2_O/rGO aerogel showed superior photocatalytic activity in MO degradation owing to the broadening light absorption capability [20]. Chen et al. [21] demonstrated that 3D Ag/Ag@Ag_3_PO_4_/GA exhibited excellent degradation efficiency of organic dye contaminants in the visible light. BiOCl, with a tunnel-like structure, has been regarded as a new type of photocatalytic or catalytic materials in recent years [22,23]. Due to the enhanced visible-light response and efficient separation of electron and hole, BiOCl/rGO aerogel, fabricated by a chemical reduction self-assembly method at 180 ℃ for 6 h, displayed an outstanding synergistic effect on the adsorption and photocatalytic degradation of oxytetracycline [24]. BiOCl/rGO aerogel was prepared via a combined hydrothermal method with vacuum freeze drying [25]. However, the combination of BiOCl using BiCl_3_ as raw material with graphene to fabricate 3D aerogel without the vacuum freeze-drying process for the treatment of MO wastewater by activating PDS has rarely been reported under simulated sunlight irradiation.

Herein, a series of BiOCl/GA hybrid architectures with different BiOCl contents were successfully synthesized via an in situ hydrothermal method followed by natural drying. The physicochemical properties and photoelectrochemical properties of BiOCl/GA composites were systematically examined by a range of characterization techniques, and the catalytic activity of BiOCl/GA composites was evaluated by activating PDS for MO degradation. The experiments under different conditions, such as BiOCl ratio, pH, PDS concentration, activator dosage, and co-existing anions, were conducted to study the influence on the removal of MO in the presence of simulated sunlight irradiation. Moreover, the recycling experiment was also used to evaluate the stability of BiOCl/GA in PDS oxidation system. In addition, a free radical capture experiment was further undertaken to identify the dominant reactive species. 

## 2. Results and Discussion 

### 2.1. Crystal Structure of Fibers of BGA-4

**Figure 1:**
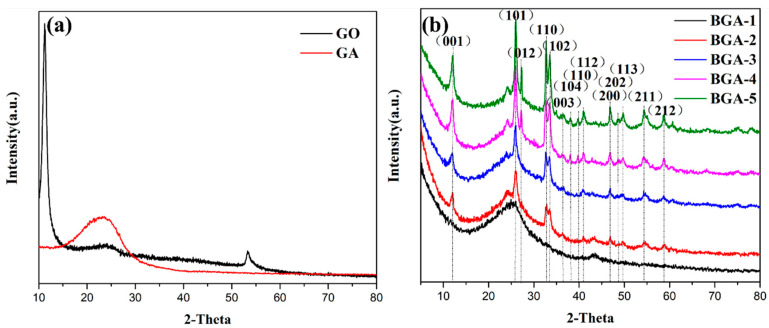
XRD patterns of GO and GA (**a**), BGA-1, BGA-2, BGA-3, BGA-4, and BGA-5 composites (**b**).

XRD is an effective method for evaluating the crystalline properties of composites. The XRD patterns of GO, GA, and BGA were illustrated in Figure 1. As presented in Figure 1a, the peak of GA appeared at about 2θ = 24°; meanwhile, the characteristic peak at 2θ = 11.6° disappeared, indicating that most oxygen-containing functional groups belongs to GO were reduced successfully. As shown in Figure 1b, the distinctive peaks at 11.9°, 25.9°, 32.5°, 33.4°, 36.5°, 40.9°, 46.6°, 49.7°, 54.1°, and 58.6° of BGA were perfectly consistent with (001), (101), (110), (102), (003), (112), (200), (113), (211), and (212) planes of the tetragonal phase of BiOCl (JCPDS 06-0249), respectively. Furthermore, the peak intensity of BGA was directly proportional to the content of BiOCl (≤15%), which indicated that the graphene had no effect on the growth orientation of BiOCl but did restrain the growth of BiOCl crystal in some degree [26]. As the content of BiOCl increases, four characteristic peaks of BGA-4 and BGA-5 at 2θ of 27.3°, 38.1°, 39.7°, and 48.7° were subordinated to (012), (104), (110), and (202) crystal planes of Bi metal (JCPDS card No. 44-1246), which demonstrated a small quantity of Bi^3+^ being reduced to metal Bi.

**Figure 2:**
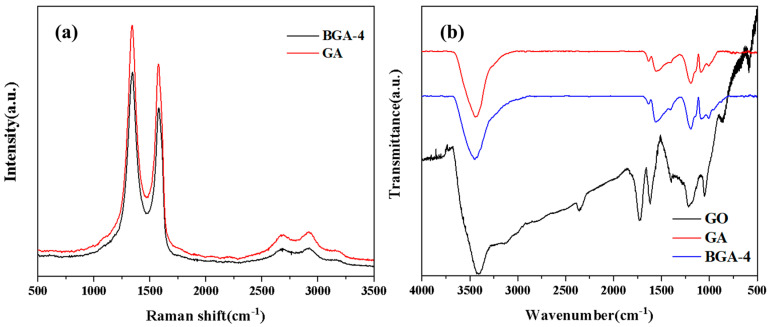
Raman analysis (**a**) of the prepared GA and BGA-4, and FTIR spectra (**b**) of the prepared GO, GA, and BGA-4.

Raman spectra were used to analyze the chemical structure of GA and BGA-4. As presented in Figure 2a, two characteristic peaks occurred at 1344 cm^−1^ and 1582 cm^−1^, which belonged to the D and G bands of graphene. The D peak, reflecting the defects and disorder of crystal, represents the sp^3^ hybrid structure or the sp^2^ bond hybrid defect graphene edge structure. The G peak is generated by the stretching motion of all sp^2^ atom pairs [27]. The ratios of D band to G band intensity (ID/IG) for GA and BGA-4 were 1.16 and 1.22, indicating that BGA-4 had a higher defects and disorders degree than GA, which suggested that BiOCl has a remarkable disruption of symmetrical lattice for BGA composites. 

FT-IR spectroscopy was used to explore the chemical structure of the GO, GA, and BGA-4. As displayed in Figure 2b, the peaks of GO at about 1059 cm^−1^, 1229 cm^−1^, 1400 cm^−1^, 1618 cm^−1^, 1734 cm^−1^, and 3419 cm^−1^ correspond to the antisymmetric and symmetric stretching vibrations of C-O-C, C-OH, O-H, C=C, carbonyl C=O, and O-H tensile vibration of H_2_O molecules absorbed to the surface, respectively [28]. These oxygen-containing functional groups became weaker or even disappeared in GA and BGA compared to the GO spectrum, confirming the efficient reduction of GO. Owing to the low content of BiOCl, the typical mode of Bi-O was far too subtle to be observed at 536.1 cm^−1^ in the spectrum of BGA [29]. 

**Figure 3:**
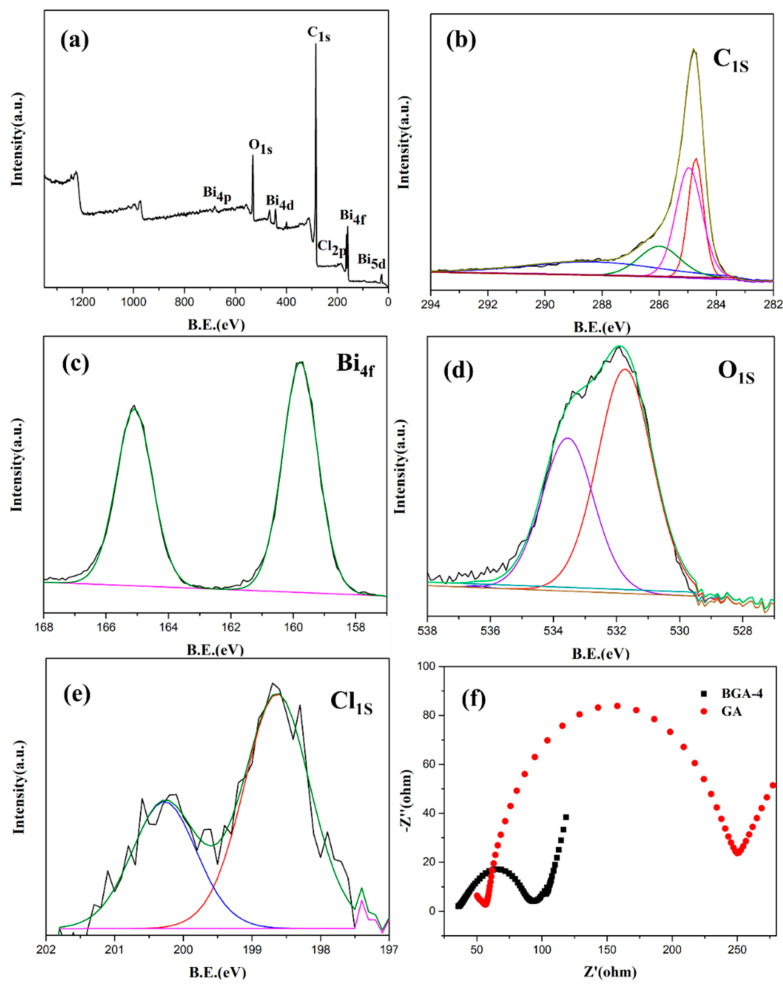
XPS spectra of the survey (**a**), C_1s_ (**b**), Bi_4f_ (**c**), O_1s_ (**d**), and Cl_1s_ (**e**) for BGA-4 composite, and EIS (**f**) of GA and BGA-4.

The elemental composition and chemical properties on the surface of the BGA-4 were determined by X-ray photoelectron spectroscopy (XPS). As reflected in Figure 3a, the peaks of Bi 5d, Bi 4f, Bi 4d, Bi 4p, Cl 2p, C 1s, and O 1s appeared in the survey scan spectra of BGA-4, which indicated that BiOCl had been successfully introduced into BGA-4. The high-resolution XPS spectra of C1s was shown in Figure 3b; the C 1s peak of BGA-4 was fitted into four peaks at about 284.6 eV, 284.9 eV, 286.0 eV, and 288.9 eV, corresponding to C-C, C-O, C-OH, and O=C-O bonds, respectively. The results indicated that some of the oxygen-containing groups on GO were retained in the process of BGA-4 synthesis, which may be beneficial for the activation of PDS. Two characteristic peaks (Figure 3c) at 159.7 eV and 165.0 eV were ascribed to Bi 4f 5/2 and Bi 4f 7/2, indicating the existence of Bi^3+^ in BGA-4 [30]. As shown in Figure 3d, the O 1s core-level spectrum of BGA-4 could be mainly divided into two peaks at about 531.5 and 533.7 eV, corresponding to the Cl–O and C–O bonds in XPS [31]. The high-resolution Cl 2p spectra (Figure 3e) including two obvious characteristic peaks at 198.6 eV and 200.2 eV were ascribed to Cl 2p 1/2 and Cl 2p 3/2, indicating the existence of BiOCl in BGA-4.

EIS was conducted to clarify the contribution of BiOCl on the conductivity and charge carrier transfer of BGA-4. As illustrated in Figure 3f, the Nyquist plots were a sort of combination of semicircles and straight lines. The arc radius of the Nyquist circle reflected the charge transfer resistance. Smaller charge transfer resistance might be conducive to the separation and transfer efficiency of the optical carrier [32,33]. The ohmic resistance (R_S_) and the charge transfer resistance (R_CT_) of BGA-4 were 39.73 Ω and 54.15 Ω, substantially lower than those of GA (42.26 Ω and 111.74 Ω), which indicated that BiOCl could improve the conductivity and electron transfer capacity of BGA-4 composite. The rapid electrolyte ion transport to the active sites and the enhanced separation of photoelectrons from vacancies might be conducive to PDS activation.

**Figure 4:**
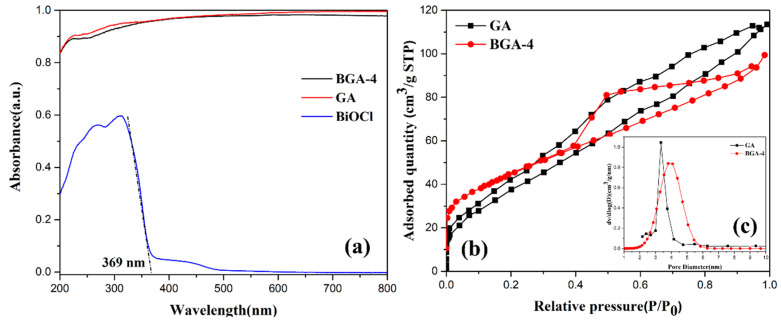
UV–Vis DRS of BiOCl, GA, and BGA-4 (**a**), N_2_ adsorption–desorption isotherms (**b**), and pore size distribution plots (**c**) of GA and BGA-4.

The UV–Vis DRS of the pristine BiOCl, GA, and BGA-4 are exhibited in Figure 4a. It can be seen that BiOCl possesses a steep absorption edge at about 369 nm, which was consistent with the band gap (3.36 eV) estimated based on the formula of Eg = 1240/λ_g_ (eV). However, GA and BGA-4 demonstrated strong absorption in the whole visible light region because of the surface sensitization and black-body property of aerogel [34]. The strong capacity of light absorption was conducive to remove MO by photo-assisted activation of PDS.

The BET-specific surface area and pore diameter of GA and BGA-4 determined by N_2_ adsorption–desorption isotherms were reflected in Figure 4b,c. Judging from the new classification method for physical adsorption isotherms formulated by the International Union of Pure and Applied Chemistry (IUPAC), both GA and BGA-4 displayed the typical type-IV isotherms with clear H3 hysteresis loop (Figure 4b). The BET of GA and BGA-4 were calculated to be 153.12 m^2^·g^−1^ and 158.76 m^2^·g^−1^, respectively. The introduction of BiOCl slightly increased the surface area of BGA-4, which was consistent with the results of Zhang et al. [24]. According to Figure 4c, the main pore size distribution of BGA-4 (3.94 nm) was slightly greater than GA (3.35 nm). The greater pore diameter may be caused by the interaction between 3D BiOCl and RGO. It can be seen that the prepared activators belonged to typical mesoporous material. The porous structure and high specific surface area of BGA-4 are favorable for light capture, charge transport, and mass transfer, and thus to advance the oxidative degradation of MO by PDS under simulated sunlight [35].

**Figure 5:**
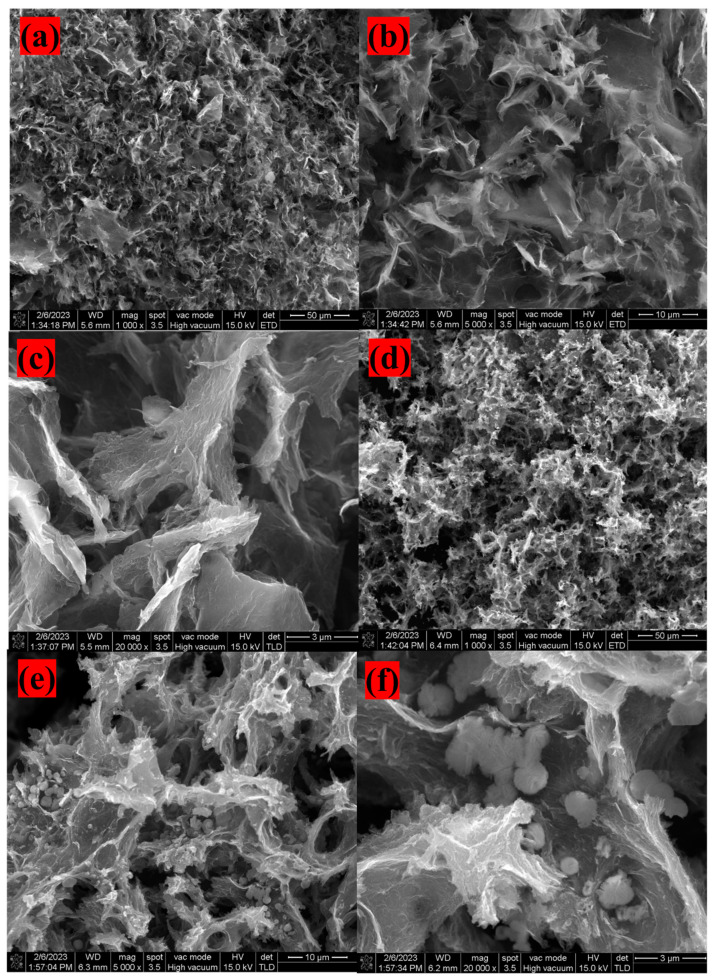
SEM images of GA (**a**–**c**) and BGA-4 (**d**–**f**).

SEM was exploited to characterize the morphology of the prepared GA and BGA-4. Figure 5a–c represented that GA still exhibited the layered structure of graphene sheets and then folded into a multiporous network structure. As displayed in Figure 5d–f, the SEM images of BGA-4 showed that the 3D flower-like uniformly microspheres (1–2 μm) were successfully doped into the surface or internal cavities of BGA-4, indicating the successful synthesis of BGA-4 using GO and BiCl_3_ as raw materials. 

**Figure 6:**
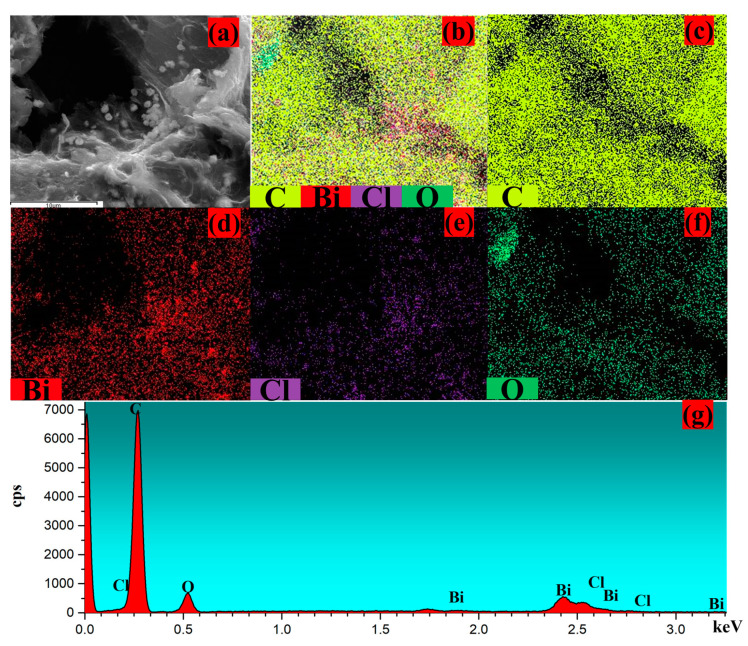
SEM image (**a**), elemental mapping (**b**–**f**), and EDS spectra (**g**) of BGA-4.

The EDS results (Figure 6) of BGA-4 clearly demonstrated the presences and spatial distributions of C, Bi, Cl, and O elements (Figure 6c–f), further demonstrating the successful hybridization of BGA-4. The content of major elements (C, Bi, Cl, and O) was 76.50%, 7.44%, 0.8%, and 15.26%, respectively. The atomic number ratio of Bi/Cl was 1.6:1, which demonstrated that most bismuth existed in the form of BiOCl, and perhaps other forms exist. The results are consistent with findings in XRD patterns, as illustrated in Figure 1.

### 2.2. Comparative Tests on MO Removal

**Figure 7:**
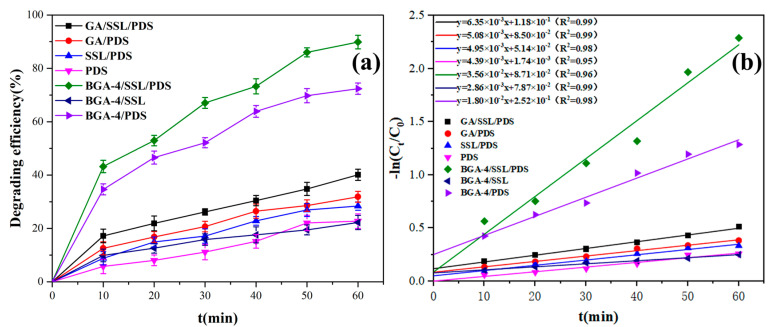
MO decontamination efficiency (**a**) and pseudo-first-order plots (**b**) of MO degradation over various processes (reaction conditions: 0.4 g/L catalyst, 75 mg/L PDS, 300 W xenon lamp, and C_0_ = 15 mg/L).

The batch experiments on MO degradation were implemented under simulated sunlight and PDS oxidative environment, respectively. As illustrated in Figure 7a, MO loss by PDS (22.8%), GA/PDS (31.8%), SSL/PDS (28.4%), and GA/SSL/PDS (40.1%) systems were both less than 50% within 60 min, which indicated that PDS was not easily activated by simulated sunlight, but could be decomposed to produce SO_4_**·**^−^ by the lone pairs of electrons belonging to C=O of GA [36]. Meanwhile, it can be observed that the removal efficiency of MO was 40.1%, 22.2%, 72.4%, and 89.9% in the GA/SSL/PDS, BGA-4/SSL, BGA-4/PDS, and BGA-4/SSL/PDS systems, respectively. The results revealed that graphene aerogel composites decorated with BiOCl would expose more active sites on the catalyst surface to the target contaminant. Moreover, the decontamination of MO may also be ascribed to the photo-induced e^-^/h^+^ pairs to SSL irradiation and the efficient electron transfer and adsorption of both PDS and the contaminant onto active sites in the BGA-4/SSL/PDS system [37,38]. As shown in Figure 7b, the MO removal behavior in vitro followed the first order kinetic equation (R^2^>0.95). The apparent reaction rate constants (K_obs_) for MO degradation were 6.35 × 10^−3^ min^−1^ (GA/SSL/PDS), 5.08 × 10^−3^ min^−1^ (GA/PDS), 4.95 × 10^−3^ min^−1^ (SSL/PDS), 4.39 × 10^−3^ min^−1^ (PDS), 3.56 × 10^−2^ min^−1^ (BGA-4/SSL/PDS), 2.86 × 10^−3^ min^−1^ (BGA-4/SSL), and 1.80 × 10^−3^ min^−1^ (BGA-4/PDS) in different systems, respectively. The experiment results demonstrated that the MO degradation rate by BGA-4 composite was 5.61 times that of GA in PDS activation under simulated sunlight irradiation.

### 2.3. Parameters Impacting MO Degradation by the BGA-4/SSL/PDS System 

#### 2.3.1. Effect of the Catalyst BGA-4 Concentration

**Figure 8:**
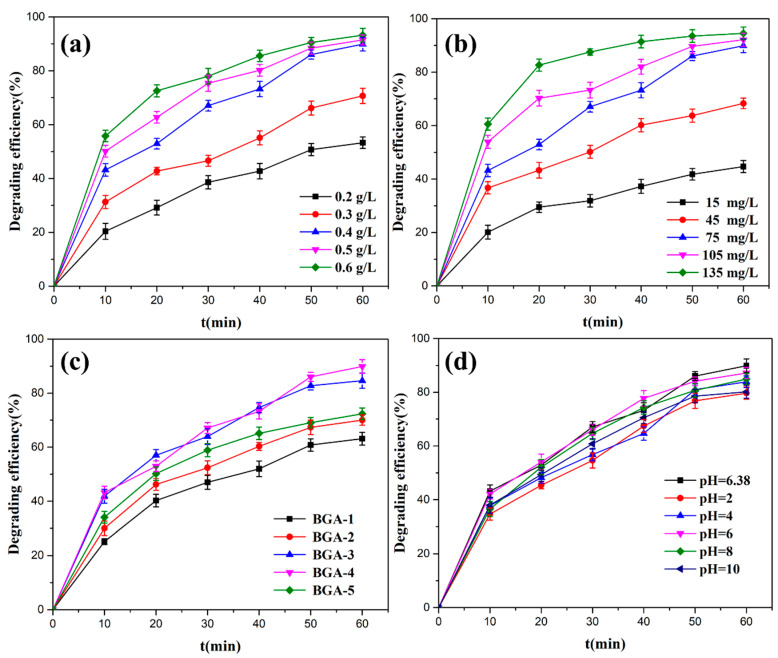
Effect of BGA-4 dosage (**a**), PDS concentration (**b**), BiOCl doping amount (**c**), and initial pH (**d**) on the degradation of MO.

BGA-4 dosage exhibited a significant effect on MO removal (Figure 8a). When the dosage increased from 0.2 g/L to 0.6 g/L in the BGA-4/SSL/PDS system, the removal efficiency of MO was 53.3% (0.2 g/L), 70.7% (0.3 g/L), 89.9% (0.4 g/L), 91.5% (0.5 g/L), and 92.3% (0.6 g/L) within 60 min, respectively. The degradation efficiency was significantly enhanced with the BGA-4 concentration increasing from 0.2 g/L to 0.4 g/L, which might be attributed to the enhanced proteolytic activity and surface area for the effective activation of PDS. However, the degradation efficiency of MO increased slowly at higher dosages (>0.4 g/L), suggesting that PDS played an important role in MO degradation when the activator was sufficient. Thus, the concentration of activator was set to 0.4 g/L for the follow-up experiments.

#### 2.3.2. Effect of PDS Concentration

The effect of PDS concentration on MO degradation was evaluated in Figure 8b over the BGA-4/SSL/PDS system. The removal efficiency of MO was 44.7%, 68.3%, 89.9%, 92.1%, and 94.5%, when the PDS concentration was set to 15 mg/L, 45 mg/L, 75 mg/L, 105 mg/L, and 135 mg/L, respectively. The removal efficiency of MO increased from 44.7% to 94.5% within 60 min, indicating that it is advantageous to add PDS in MO degradation. The reason may be that high concentration of PDS generated more active species in the reaction system. PDS acted as electron acceptor could reduce O_2_ to O_2_**·**^−^ [39], and O_2_**·**^−^reacted with some of PDS to form SO_4_**·**^−^ [40]. While the concentration of PDS progressively increased from 75 mg/L to 135 mg/L, the degradation rate of MO leveled off, perhaps related to a finite number of photoelectrons. After comprehensive consideration, the optimal concentration of PDS was selected as 75 mg/L.

#### 2.3.3. Effect of BiOCl Doping Amount

The effect of BiOCl concentration in aerogel composites on MO degradation was exhibited in Figure 8c. BGA-4 displayed the highest degradation efficiency of 89.9%. As the corresponding mass ratio of BiOCl increased from 5% to 20%, the degradation efficiency of MO increased from 63.1% to 89.9%. The reason might be that more BiOCl can produce more photoelectrons and holes, thus promoting the activation of PDS to produce more free radicals. It was worth noting that if the amount of BiOCl further increased to 25%, the degradation efficiency of MO dropped to 72.3%. The result implied that BiOCl content was a key factor for improving the photocatalytic activity of aerogel composites [41]. Therefore, the optimal mass ratio of BiOCl for this study was set at 20%.

#### 2.3.4. Effect of pH

The photocatalytic degradation of MO was greatly influenced by the solution pH in the BGA-4/SSL/PDS system. As displayed in Figure 8d, the removal efficiencies of MO were 79.7% (pH = 2), 83.9% (pH = 4), 87.2% (pH = 6), 84.9% (pH = 8), and 80.2% (pH = 10), respectively. Higher or lower pH of the solution was detrimental to the degradation efficiency of MO, which might be attributed to the scavenging effect of H^+^ on the activity factor at lower pH and less efficiency in the generation of the reactive species at higher pH [38], while the highest degradation rate of MO was obtained at pH 6. In general, BGA-4 exhibited a high removal rate of MO across a broad range of pH in the BGA-4/SSL/PDS system. Since the pH of the MO solution (6.23) is closer to 6, the whole experiment is carried out without pH adjustment. 

### 2.4. Effect of Co-Existing Components

**Figure 9:**
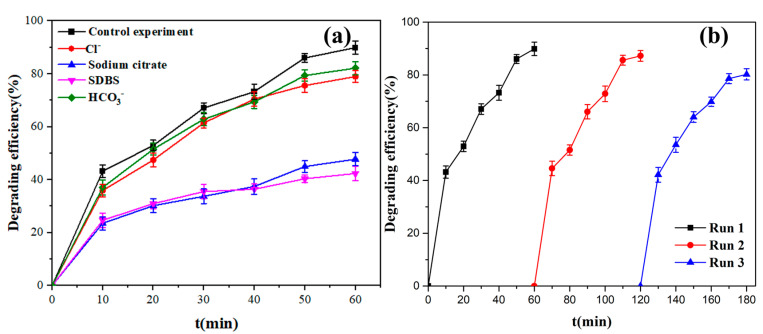
Effect of coexisting ions (**a**) and the reusability potential (**b**) of as-prepared catalyst in the BGA-4/SSL/PDS system (reaction conditions: 0.4 g/L BGA-4, 75 mg/L PDS, 300 W xenon lamp, and C_0_ = 15 mg/L).

In practical wastewater, the presence of inorganic anions printing and textile auxiliaries, such as HCO_3_^−^, Cl^−^, citrate, and sodium dodecyl benzene sulfonate (SDBS), is obvious. Usually, these co-existing anions showed inhibition towards the removal of MO in the following ways by rapidly reacting with the free radicals and by changing the acidic/basic conditions [42]. As displayed in Figure 9a, the degradation of MO was inhibited by the co-existing anions (10 mM) to varying degrees. The presence of HCO_3_^−^ (82.1%) had a passive effect on the degradation of MO, probably because the increased pH that could restrain the generation of activity factors. The removal rate of MO fell to 47.7% because of the h^+^ captured by sodium citrate in the BGA-4/SSL/PDS system. Generally, Cl^−^ could react with SO_4_**·**^−^ or ·OH to form Cl·, whose oxidation susceptibility is relatively weak. The generation of O_2_·^−^ was suppressed because of the dissolved oxygen reduced by the bubbles generated from SDBS, thus causing the removal ratio of MO to be pared down to 42.3%.

### 2.5. Recycling Studies

The recycling and stability of BGA-4 for PDS activation with the optimized dosages was evaluated through three cycles of experiments. The used BGA-4 was washed simply with deionized water and ethanol 2−3 times before each experiment started. As shown in Figure 9b, the MO removals in three experiments were 89.9%, 87.2%, and 80.2% in the BGA-4/SSL/PDS system, respectively. The degradation rate of MO decreased because the active sites reduced by MO and byproducts remained on the surface of BGA-4 via van der Waals interaction force [43]. The experimental results demonstrated that BGA-4 still had good stability and removal ability after three cycles. Table 1 displays a comparison of efficiency regarding the removal of MO by PDS activation systems in this work and several reported relative studies, which indicated that the BGA-4/SSL/PDS system is a potential approach for dyeing wastewater treatment.

### 2.6. Role of Reactive Oxidizing Species in the BGA-4/SSL/PDS System

**Figure 10:**
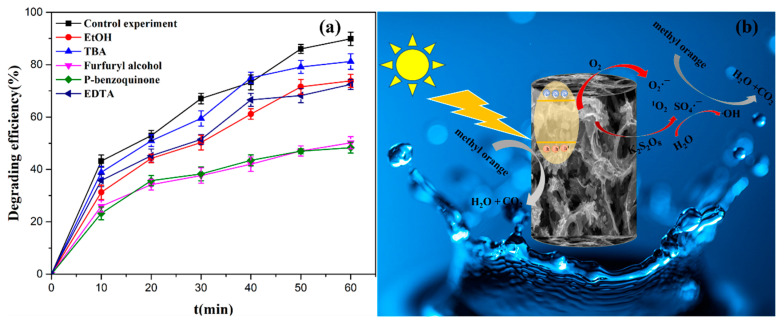
The effect of quenching agents (20 mM) on the degradation efficiency of MO (**a**) and the schematic mechanism of MO degradation (**b**) in the BGA-4/SSL/PDS system (reaction conditions: 0.4 g/L BGA-4, 75 mg/L PDS, 300 W xenon lamp, and C_0_ = 15 mg/L).

A chemical quenching experiment was performed to investigate the dominant reactive species in the BGA-4/SSL/PDS system. Initially, ethanol (EtOH, 20 mM), tert-butanol (TBA, 20 mM), p-benzoquinone (20 mM), ethylene diamine tetraacetic acid (EDTA), and furfuryl alcohol (20 mM) were employed as the radical scavengers of SO_4_·^−^, ·OH, O_2_·^−^, h^+^, and ^1^O_2_ to the BGA-4/SSL/PDS system. As shown in Figure 10a, the degradation rates of MO were 73.8%, 81.2%, 48.3%, 72.4%, and 50.2% after 60 min, respectively. EtOH, TBA, and EDTA only showed 16.1%, 8.7%, and 17.5% inhibition in MO degradation, respectively, suggesting the insignificant role of free radicals (SO_4_·^−^, ·OH, and h^+^). In addition, furfuryl alcohol and p-benzoquinone caused the degradation rate of MO to significantly decrease by 28.4% and 25.4%, indicating that ^1^O_2_ and O_2_·^−^played a vital role in MO degradation. ^1^O_2_ might be generated though the nucleophilic addition with the aid of the activation of C-O groups to PDS [44]. The formation of O_2_·^−^was produced through the reaction between the photo-induced electron produced by BiOCl and the dissolved oxygen under simulated sunlight irradiation. The contribution rate of free radicals on MO removal was in the sequence of ^1^O_2_ > O_2_·^−^ > h^+^ > SO_4_·^−^ >·OH in the BGA-4/SSL/PDS system.

**Table 1:** Comparison of efficiency of removing MO in similar catalytic systems.

Process	Light Source	MO Concentration	Catalyst Dosage	PDS Concentration	Degradation Efficiency	Reaction Time	Ref.
GO/PDS/ES	Tungsten-halogen lamp (250 W)	1.5 mM	6 mg·L^−1^	0.05 mM	77%	240 min	[45]
P25-TiO_2_/ZnO/PDS	Tungsten lamp (100 W)	20 mg/L	0.5 g/L	3 ppm	82%	240 min	[46]
ZnO/AgFeO_2_/PDS	Visible light (LED 50 W)	1 × 10^−5^ M	0.4 g/L	1.48 mM	70%	300 min	[47]
TiO_2_/Fe_3_O_4_/PDS	UV light (300 W)	20 mg/L	0.4 g/L	60 mM	99.5%	150 min	[48]
Fe_0_/PDS	solar	10 mg/L	100 mg/L	360 mg/L	95%	120 min	[49]
BGA-4/PDS	simulated sunlight (Xe 300 W)	15 mg/L	0.4 g/L	0.075 g/L	89.9%	60 min	This study

Multiple pathways were responsible for the MO degradation process (Equations (1)–(6)), and the schematic mechanism is shown in Figure 10b. Firstly, MO molecules were adsorbed on the surface of BGA-4 and then degraded by various reactive oxidizing species. The photo-induced electron reacted with S_2_O_8_^2−^ and the dissolved oxygen in the solution, respectively. The oxidizing species generated by the above reactions led to the degradation of MO. The rGO thin sheets in BGA-4 could prevent the recombination of photo-induced electrons (e) and holes (h^+^), which is of benefit to the removal of MO in the BGA-4/SSL/PDS system.

(1) $$\mathrm{hv}\overset{\mathrm{BGA}-4}{\to }e\;+{\;h}^{+}$$

(2) $$e\;+S_{2}O_{8}^{2-}\;\to {\;\mathrm{SO}}_{4}^{-}·+{\mathrm{SO}}_{4}^{2-}$$

(3) $${\mathrm{SO}}_{4}^{-}·+H_{2}O\;\to \;\mathrm{HO}·+{\mathrm{SO}}_{4}^{2-}+H^{+}$$

(4) $$e\;+O_{2}\;\to {\;O}_{2}^{-}·$$

(5) $$\mathrm{BGA}-4+S_{2}O_{8}^{2-}+H^{+}\;\to {}^{1}O_{2}+{\mathrm{SO}}_{4}^{2-}+H_{2}O\;$$

(6) $$\mathrm{MO}\;+{}^{1}O_{2}/O_{2}^{-}·/h^{+}/{\mathrm{SO}}_{4}^{-}·/\mathrm{HO}·\to \;\mathrm{Degradation}\;\mathrm{products}\;$$

## 3. Materials and Methods

### 3.1. Synthesis of GA and BiOCl/GA Composite

**Scheme 1:**
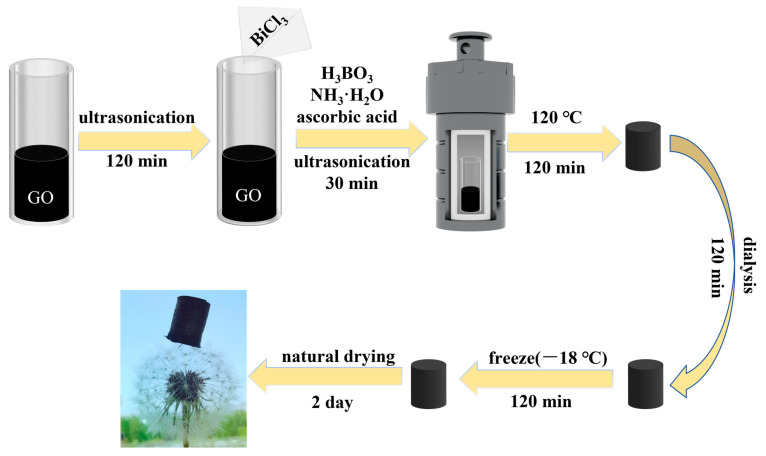
The preparation diagrammatic drawing of BGA.

GO was prepared from natural graphite powder by means of a modified Hummers method [50]. A total of 25 mg of GO powder was dispersed in 5 mL of deionized water and ultrasonically treated for 2 h. The BGA (BiOCl/GA) with different BiOCl concentrations were fabricated in situ by a simple hydrothermal method. The mass percent concentration of BiOCl in the BGA sample was 5%, 10%, 15%, 20%, and 25%, respectively. In brief, certain quantities of BiCl_3_, 38 µL NH_3_·H_2_O, 25 mg Na_2_B_4_O_7_·10H_2_O, and 50 mg ascorbic acid were added successively into GO solution and dispersed by ultrasound for 0.5 h. The homogeneous mixture was quickly poured into a Teflon-lined stainless-steel autoclave and maintained at 120 °C for 2 h. The as-formed hydrogel was immersed in 1% ethyl alcohol solution for 2 h then frozen at −18 °C for 2 h and air-dried for 48 h to obtain the ultra-light and compressible BGA. The preparation scheme of BGA was shown in Scheme 1. Different mass proportions of BGA hybrids (5%, 10%, 15%, 20%, and 25%) were named as BGA-1, BGA-2, BGA-3, BGA-4, and BGA-5, respectively. GA was synthesized by the same procedure without adding BiCl_3_.

### 3.2. Characterization

The structures of the samples were examined by X-ray diffraction (XRD, Rigaku/Smart LabSE). The morphology was evaluated by scanning electron microscopy (SEM; FEI-NOVA NANOSEM 230 (EDS X-MAX50)). X-ray photoelectron spectroscopic (XPS, Thermo Scientific ESCALAB Xi+) analyses were performed. An adsorption analyzer (BET, BELSORP MaxII) was used to calculate the specific surface area. Raman analysis was carried out at a wavelength of 514 nm (Ar-ion laser) using a Raman spectrometer (Raman, SPEX-1403). Infrared spectra were acquired with a Fourier-transform infrared (FT-IR) spectrometer (FT-IR, IRTracer 100). Electrochemical impedance spectroscopy was studied via an electrochemical system (EIS, CHI E660).

### 3.3. MO Degradation Procedure

The activation performance for PDS of the prepared GA and BGA composites towards MO solution (15 mg/L) was evaluated under simulated sunlight irradiation at ambient temperature (14 ± 1 °C). Briefly, the activator was directly put into 50 mL MO wastewater; it took 40 min to achieve an adsorption–desorption equilibrium. Afterwards, the cooling water system, the blower system, and the 300 W xenon lamp (100 mW/cm^2^) were turned on successively before PDS was added. At given reaction time intervals, the concentration of MO was estimated by a UV–Vis spectrophotometer (1901PC) according to the absorbance at 464 nm. The effects of BiOCl ratio, PDS concentration, activator dosage, initial pH of solution, and coexisting ions on the degradation of MO were investigated, respectively.

## 4. Conclusions

The multi-functional BiOCl/GA composites were successfully fabricated by the hydrothermal method and through a facile self-assemble approach. Characterization results indicated that BGA-4 composite aerogel possessed a 3D interconnected porous network structure and superior photocatalytic activity in the presence of PDS under simulated sunlight irradiation. The degradation rate constant of MO by the BGA-4/SSL/PDS system (3.56 × 10^−2^ min^−1^) was 5.61 times that of the GA/SSL/PDS system (6.35 × 10^−3^ min^−1^). The production and rapid electron transfer of photogenic electrons played a vital role in MO removal in the BGA-4/SSL/PDS system. The quenching experiments affirmed that the contribution of reactive species was in a sequence of ^1^O_2_ > O_2_·^−^ > h^+^ > SO_4_·^−^ >·OH. The catalyst BGA-4 also exhibited excellent stability, high separation capability, and reusability potential. Therefore, the present study not only illustrated an in situ synthesis method for 3D macrostructure BGA-4 but also presented a potential BGA-4/SSL/PDS system for the highly efficient treatment of waste dyeing water.

## Data Availability

Not applicable.

## Sample Availability

Samples are available from the author.
